# Development of New Chloroplast Microsatellites for *Pinus gerardiana* and their Application in Genetic Diversity Analyses

**DOI:** 10.1002/ece3.71185

**Published:** 2025-04-01

**Authors:** Sayed Jalal Moosavi, Markus Mueller, Oliver Gailing

**Affiliations:** ^1^ Forest Genetics and Forest Tree Breeding University of Göttingen Göttingen Germany

**Keywords:** chloroplast genome, cpSSRs, gene flow, pollen dispersal, population structure

## Abstract

Chloroplast DNA (cpDNA) is a valuable tool for studying plant population genetics and gene flow by pollen in conifers due to their paternal inheritance, particularly through the application of chloroplast DNA microsatellite markers (cpSSRs). This study focuses on Chilgoza pine ( *Pinus gerardiana* L.), an economically and ecologically significant tree species in Afghanistan. Despite its importance, comprehensive genetic research on Chilgoza pine has been limited. To address this gap, we developed novel cpSSR markers based on the Chilgoza pine's chloroplast genome to assess genetic diversity, population structure, and pollen dispersal in a population from Gardiz, Afghanistan. Needle samples from 199 trees across four subpopulations and two age cohorts (young and old) were collected and analyzed. Our findings revealed 27 chloroplast microsatellite markers, of which six exhibited polymorphism. Haplotype analysis identified 32 unique haplotypes, with one most prevalent haplotype. Genetic diversity analysis showed comparatively high levels of diversity, with no genetic differentiation between young and old tree cohorts. Fine‐scale spatial genetic structure (FSGS) analysis revealed significant but weak family structure and relatedness in young cohorts, suggesting distance‐dependent gene flow by pollen. Additionally, in silico BLAST analysis demonstrated strong sequence conservation across related *Pinus* species, indicating high potential for cross‐species amplification of the developed markers. Our study underscores the utility of cpSSRs in characterizing genetic diversity and structure, which is crucial for the conservation and sustainable management of Chilgoza pine forests. These findings provide insights for developing conservation strategies and highlight the importance of genetic marker studies to aid the preservation of biodiversity that supports local livelihoods.

## Graphical abstract

This study developed novel chloroplast DNA microsatellite markers (cpSSRs) to assess genetic diversity, population structure, and pollen dispersal in Chilgoza pine ( *Pinus gerardiana* ) from Gardiz, Afghanistan. We identified 32 unique haplotypes and observed high genetic diversity, weak family structure, and no differentiation between tree age cohorts. These findings provide critical insights for the conservation and sustainable management of this ecologically and economically vital species.

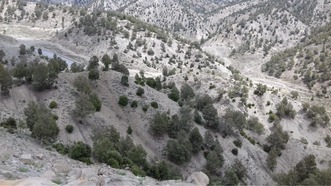

## 1 Introduction

To unravel the complexities of plant population structure and genetic variation, the chloroplast (cp) genome serves as an invaluable resource (Daniell et al. 2016; Provan et al. 2001). The chloroplast genome is paternally (via pollen) inherited in conifers and does not recombine (Powell et al. 1995a). Thus, cp‐based molecular markers are instrumental for the study of genetic diversity and structure, and gene flow via pollen and its contribution to population connectivity and structure (Dzialuk et al. 2009; Powell et al. 1995a; Terrab et al. 2006).

In Afghanistan, the Eastern forest complex (EFC) stands out for its rich natural resources, diverse geography, and rugged topography. Among these resources, *Pinus gerardiana* L. (Chilgoza pine) holds particular significance (Shalizi et al. 2016). This species thrives in arid valleys spanning eastern Afghanistan, neighboring regions of northern and northwestern Pakistan, northwestern India, and areas within Tibet and the Xizang province of China. Despite its value, the Chilgoza pine faces increasing pressures, highlighting the urgent need for more comprehensive and targeted conservation strategies (Bonner and Karrfalt 2008; Critchfield and Little 1966).

*Pinus gerardiana* thrives in semi‐arid mountains at 1800–3350 m a.s.l., stabilizing soils and supporting wildlife through its seeds (Farjon 2010). It relies on *Nucifraga multipunctata* (large‐spotted nutcracker‐unknown abundance in the study region) for seed dispersal, crucial for forest regeneration (Lanner 1990; Madge et al. 2020). *P. gerardiana* belongs to subsection *Gerardianae* (including *P. gerardiana* , *P. squamata* , and *P. bungeana* ) and is most closely related to *P. squamata* , with an estimated divergence time of 13.69 million years ago based on chloroplast DNA sequences (Zhang et al. 2024). Chilgoza pine forests play a vital role in Afghanistan's ecosystem, providing habitat for numerous wildlife species and contributing to soil conservation. However, these forests are facing increasing pressure due to overexploitation for their valuable seeds (Khurram et al. 2023), which are harvested for their culinary and medicinal uses. Furthermore, habitat fragmentation and the lack of natural regeneration, driven by unsustainable harvesting practices and grazing pressure, exacerbate their vulnerability.

To address these threats, several initiatives have been launched. Since 2001, the Afghan government and NGOs have implemented small‐scale reforestation projects, despite challenges like insecurity and limited planting stock (Groninger 2012). The Ministry of Agriculture, Irrigation and Livestock (MAIL) restored 320 ha of Chilgoza pine forests in 9 provinces and produced 40,000 saplings in Paktia, Paktika, and Khost. Additionally, 1076 household nurseries were established to produce 6,456,000 saplings for restoring 8070 ha of degraded forests of Chilgoza and walnut in these provinces (MAIL 2020).

Despite the growing awareness of its near‐threatened status in recent years (IUCN Red List version 3.1), research on the genetic diversity of this species, especially within Afghan populations, remains scarce. While there has been some research on *P. gerardiana* , much of the focus has been on ecological distribution, medicinal use, and conservation efforts, with limited attention given to genetic diversity. Specifically, only two studies on genetic diversity in Afghan populations were published (Moosavi et al. 2022; Motieimehr et al. 2023). There remains a significant gap in understanding the fine‐scale spatial genetic structure (FSGS) and how environmental factors influence genetic variation within Afghan populations. This gap highlights the need for the development of new genetic tools, such as chloroplast microsatellites, to explore the genetic diversity of *P. gerardiana* .

Previous research on the closely related conifer species, *Pinus bungeana*, has revealed low nucleotide diversity in both chloroplast and mitochondrial DNA (Zhang et al. 2024). If similar patterns are observed in *P. gerardiana* , this could indicate historical demographic events or limited genetic exchange.

The application of these microsatellites will allow for more accurate analyses of genetic variation, offering valuable insights into the species' population structure, which can ultimately inform more effective conservation strategies and enhance the species' resilience to future environmental pressures. The sustainable management of Chilgoza pine forests is crucial not only for preserving biodiversity but also for ensuring the continued livelihood of local communities dependent on these forests for their sustenance and income (Shalizi et al. 2018).

In our previous study on Chilgoza pine in Gardiz using eight Expressed Sequence Tag (EST)‐SSRs, we found a moderate level of genetic variation (mean expected heterozygosity, *H*_*e*_ = 0.344) in contrast to other pine species (Moosavi et al. 2022). Here, we developed a novel set of cpSSR markers based on the chloroplast genome of *P. gerardiana* (Cronn et al. 2008) to compare the contribution of gene flow by pollen and seed to the population genetic structure (Powell et al. 1995a).

We hypothesize that pollen‐mediated gene flow in *P. gerardiana* populations in Gardiz, Afghanistan, due to the small sampling area and close proximity of individuals, will result in low genetic differentiation between young and old cohorts and among subpopulations. We predict that FSGS will reveal a weak, yet significant, family structure in the young cohort, indicating distance‐dependent gene flow.

## 2 Materials and Methods

### 2.1 Sampling Area

**FIGURE 1:**
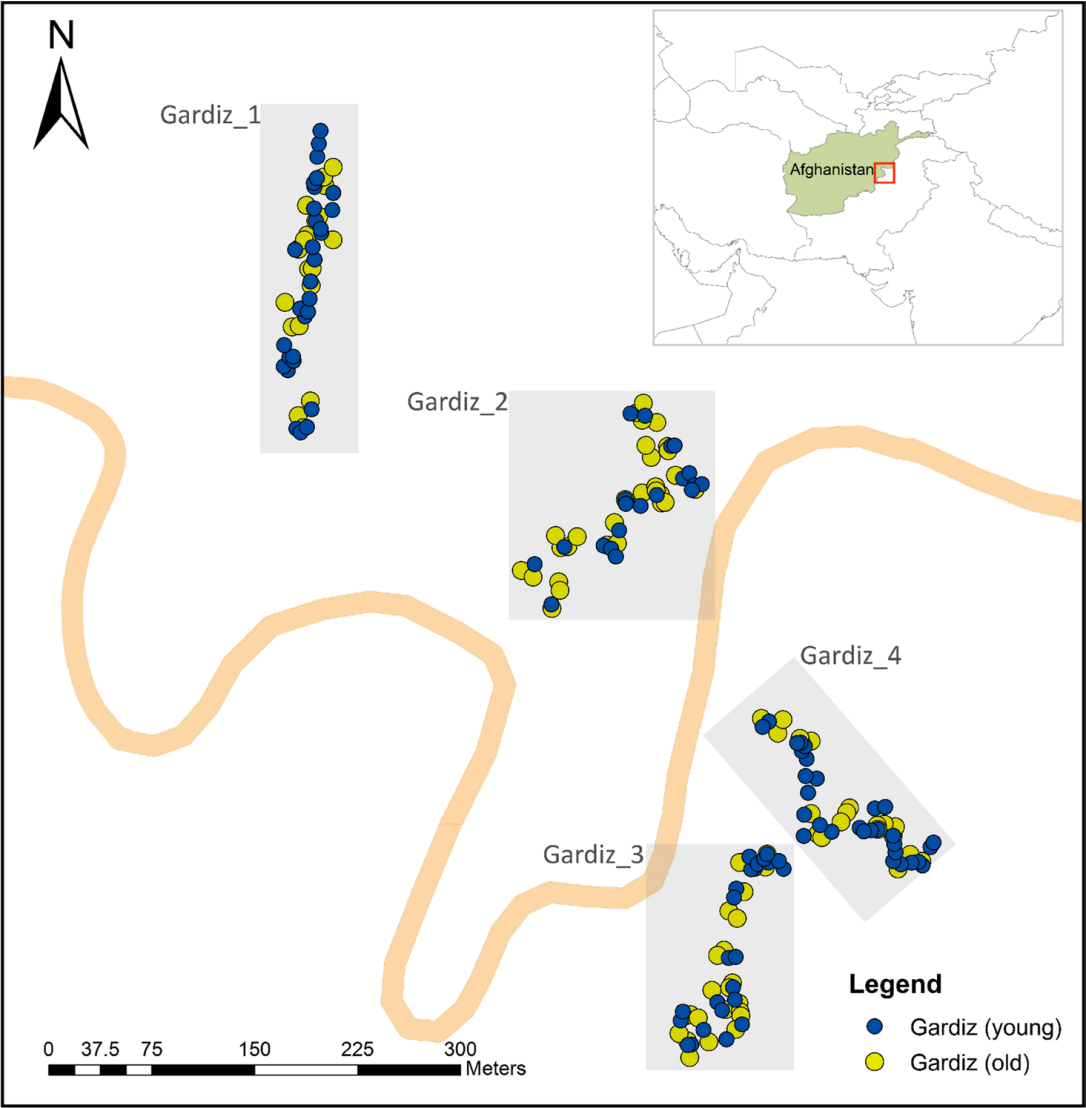
Location of each *Pinus gerardiana* individual sampled in the eastern forest complex, Gardiz, Afghanistan, adjacent to national highway 12 (NH12). Separate subpopulations (Gardiz_1–Gardiz_4) are highlighted in gray (Map was created using ArcMap v.10.8 software (Esri, USA)).

We collected Chilgoza pine needle samples from a total of 199 trees across four subpopulations (Gardiz_1, Gardiz_2, Gardiz_3, and Gardiz_4) proximate to Gardiz city, Afghanistan (Table 1, Figure 1, and Figure S1). We received verbal authorization from local authorities in Paktia province to conduct the sampling. The entire sampling area encompassed a radius of approximately 400 m. Given the mountainous terrain surrounding the *P. gerardiana* forests, the potential for pollen flow from outside populations could be limited by natural barriers, such as mountain ranges (Markgraf 1980). This geographical feature may reduce the distance over which pollen can travel, potentially leading to restricted gene flow and influencing the genetic structure of the population.

**TABLE 1:** Characteristics of the four subpopulations in the *Pinus gerardiana* population close to Gardiz, Afghanistan.

Subplots	Number of samples (Young/Old %)	Mean altitude (m)	Geographical coordinate	Mean DBH (cm)	Area (hectare)
Gardiz_1	50 (62/38)	2820.08	33°28′49.6″ N, 69°23′05.6″ E	29.79	1.26
Gardiz_2	49 (43/57)	2714.86	33°28′40.7″ N, 69°23′12.6″ E	33.36	1.00
Gardiz_3	50 (46/54)	2676.08	33°28′30.2″ N, 69°23′14.0″ E	31.22	1.08
Gardiz_4	50 (62/38)	2636.48	33°28′33.9″ N, 69°23′18.0″ E	29.94	1.22

The collected needles were dried in silica gel. Given the prevailing security concerns in many forest regions, the selection of the sampling area was contingent upon considerations of both security and accessibility.

Coordinates for each individual were recorded using a GPS device (GPSmap 60CSx, Garmin, USA). Furthermore, the diameter at breast height (DBH at 1.40 m) of each individual was measured, allowing for the classification of the population into two distinct cohorts: young (106 individuals, DBH = 6.36–28.65 cm) and old (93 individuals, 29.28–65.57 cm) individuals (Moosavi et al. 2022).

### 2.2 DNA Extraction and cpSSR Development

Genomic DNA was extracted from silica gel‐dried needles using the DNeasy 96 Plant Kit (Qiagen, Germany) following the manufacturer's instructions. DNA quality and quantity were assessed by electrophoresis in 1.0% agarose gels stained with Roti Gelstain (Carl ROTH, Germany). The isolated DNA was used directly for PCR amplification without dilution.

The chloroplast genome sequence of *P. gerardiana* was obtained from the GenBank (http://www.ncbi.nlm.nih.gov) database under accession number NC_011154. Using MISA (MIcroSAtellite) (Beier et al. 2017; Thiel et al. 2003), a bioinformatics tool for microsatellite identification, we screened the chloroplast genome for microsatellite regions. The settings used were as follows: unit size/minimum number of repeats: (1/10) (2/6) (3/5) (4/5) (5/5) (6/5). Primer‐BLAST was used to design the PCR primer pairs and OligoCalc (Kibbe 2007) to check for possible secondary structures (primer dimers and hairpins). The primer design parameters were set to default values. A total of 27 primer pairs were designed based on the identified microsatellite regions. PCRs were performed with 5′‐tailed fluorescent (6‐FAM)‐labeled M13 (5′‐CACGACGTTGTAAACGAC‐3′) forward (Kubisiak et al. 2013; Schuelke 2000) and PIG‐tailed (5′‐GTTTCTT‐3′) reverse primers (Brownstein et al. 1996).

For all primer pairs, the following touch‐down PCR program was used. The PCR started with an initial denaturation at 95°C for 15 min, followed by 10 cycles including a denaturation at 94°C for 1 min, an annealing at 60°C for 1 min, and an extension at 72°C for 1 min. After each cycle, the annealing temperature decreased by 1°C. Subsequently, 25 additional cycles (with the same denaturation and extension time and temperature) were conducted, with the annealing temperature set at 50°C for 1 min.

The PCR mixture consisted of 1.0 μL genomic DNA (approximately 0.6 ng/μL), 1.5 μL 10x reaction buffer B (Solis BioDyne, Estonia), 1.5 μL MgCl_2_ (25 mM), 1.0 μL dNTPs (2.5 mM each), 0.2 μL HOT FIREPol *Taq* DNA polymerase (5 U/μL, Solis BioDyne, Estonia), 0.2 μL tailed (Kubisiak et al. 2013; Schuelke 2000) forward primer (5 picomole/μL), 0.5 μL PIG‐tailed (Brownstein et al. 1996) reverse primer (5 picomole/μL), 1 μL (5 picomole/μL) dye labeled (6‐FAM or HEX) M13 primer, and HPLC grade H_2_O to reach a total volume of 13 μL.

The PCR products were separated using capillary electrophoresis on the ABI Genetic Analyzer 3130xl (Applied Biosystems, USA), employing GENESCAN ROX 500 as the internal size standard. This method allows for precise sizing of the PCR products. The visualization and analysis of the separated products were performed using GeneMapper 4.1 (Applied Biosystems, USA), which enabled accurate identification of alleles based on size.

A total of 27 chloroplast microsatellite markers were successfully designed for *P. gerardiana* based on its chloroplast genome sequence. Most of the SSRs were mononucleotide repeats (24 markers), with the remainder comprising two dinucleotide markers and one tetranucleotide marker. Mononucleotide repeats are commonly observed in pine species (Ni et al. 2018; Powell et al. 1995b). Although these motifs have been reported to exhibit polymorphism in some *Pinus* species, the level of polymorphism can vary depending on the specific repeat and species studied (Powell et al. 1995b).

To investigate the cross‐species amplification potential of the developed cpSSR markers for *P. gerardiana* , an in silico BLAST analysis was performed. This analysis aimed to identify sequence similarity with related species within the genus *Pinus*.

The search was conducted using the NCBI BLAST tool (https://blast.ncbi.nlm.nih.gov; Altschul et al. 1990), with the query sequences restricted to the genus “Pinus (taxid:3337)” to ensure comparisons with closely related species. The nucleotide collection (nr/nt) database was used to maximize the coverage of publicly available chloroplast genome sequences. The analysis focused on evaluating % Identity, E‐values, and alignment patterns to determine the conservation and cross‐species utility of each cpSSR marker. Special attention was given to markers showing high identity and low E‐values, as these indicate potential for cross‐species amplification.

### 2.3 Genetic Diversity and Structure Analysis

Allele size analysis was performed using GeneMapper version 4.1 (Applied Biosystems, Foster City, CA, USA). Genetic diversity estimates for each marker, including total gene diversity (*H*_*t*_), were calculated using R statistical software (v4.2.2; R Core Team 2022) with the hierfstat package (Goudet and Jombart 2022). For each marker, number of alleles (***N***_***a***_), number of effective alleles (***N***_***e***_), and Shannon Index (***I***) were calculated using GenAlEx v.6.5 (Peakall and Smouse 2006) with an input file based on fragment sizes (Breidenbach et al. 2019). Each chloroplast haplotype was characterized by the combination of cpSSR fragments. Haplotypes based on all six chloroplast markers and their frequencies were determined using the Haplotype Analysis version 1.05 software (Eliades and Eliades 2009), excluding individuals with missing data. In addition, number of haplotypes detected in each population (*A*), number of private haplotypes (*P*), effective number of haplotypes (*N*_*e*_), haplotype richness (*R*_*h*_), haplotype diversity (*H*_*d*_), and also proportion of total genetic differentiation (*F*_*st*_) were calculated using Haplotype Analysis version 1.05 software (Eliades and Eliades 2009). To assess genetic differentiation among subpopulations, we also calculated Jost's D (Jost 2008), which provides a measure of genetic differentiation that complements *F*_*st*_.

To analyze FSGS based on cpSSRs using SPAGeDi 1.5d (Hardy and Vekemans 2002), we computed Loiselle kinship coefficients *F* for sample pairs and regressed them on the logarithm of spatial distances. Significance was determined by comparing the regression slope *b*_F_ with its distribution from 10,000 permutations. We evaluated FSGS strength as *Sp* = −*b*_F_/(*F*_*1*_–1), where *b*_F_ is the regression slope and *F*_*1*_ is the mean kinship coefficient of pairs of individuals belonging to the first distance class. We excluded parent–offspring pairs by estimating FSGS separately for young and old cohorts and additionally, we calculated FSGS for the whole sample set as one population. We applied 10 to 500 m intervals for distance classes to ensure the appropriate number of individual pairs in each distance class.

## 3 Results

### 3.1 Haplotype Analysis

We tested four samples from different locations to assess amplification and polymorphism. Out of the 27 markers examined (Table S1), all but one successfully amplified. Six markers, PGCP_2, PGCP_5, PGCP_16, PGCP_18, PGCP_21, and PGCP_22, displayed polymorphisms and were subsequently used to screen the samples (Table 2). Notably, all polymorphic markers were mononucleotide repeats, although PGCP_5 was identified as a compound SSR containing a non‐repetitive sequence ((A)_12_tatactcggg(A)_12_) (Table 2).

**TABLE 2:** Six novel polymorphic chloroplast SSR markers developed in *Pinus gerardiana* .

cpSSR	Repeat motif	Primer sequence (5′–3′)	*T_m_* (C°)	Allele size range (bp)
PGCP_2	(A)_24_	F: [M13] TTATCCATCGGCCCAGTTCC	60	205–209
		R: [PIG‐TAIL] CGATCTTTTGTCCAACCAACCC	60	
PGCP_5	(A)_12_tatactcggg(A)_12_	F: [M13] TCACTTGTCAGTTCTGTCCGA	59	197–199
		R: [PIG‐TAIL] CCTTATGCTCCGTCTCACCC	60	
PGCP_16	(T)_14_	F: [M13] CCGGAGAATACAGGGCGTTA	59	355–362
		R: [PIG‐TAIL] TTCCGCATATTCCCCTTCCG	60	
PGCP_18	(T)_16_	F: [M13] TGAGTGTGAGAGGAGAGGGAA	60	229–232
		R: [PIG‐TAIL] GGTTTTCAAGACCGGAGCCA	61	
PGCP_21	(T)_14_	F: [M13] CGAAATGGTCGGAACGAATCA	59	310–314
		R: [PIG‐TAIL] GCCACAAACCCCTTTGGGAT	61	
PGCP_22	(T)_11_	F: [M13] GCGCAGTATGGGTCTAGCTT	60	208–210
		R: [PIG‐TAIL] AACCCGCAGATACAGGCAAA	60	

Abbreviation: *T*_*m*_, melting temperature.

The limited number of polymorphic markers observed might be attributed to the sampled subpopulations' close geographic proximity, which could lead to reduced genetic differentiation.

**FIGURE 2:**
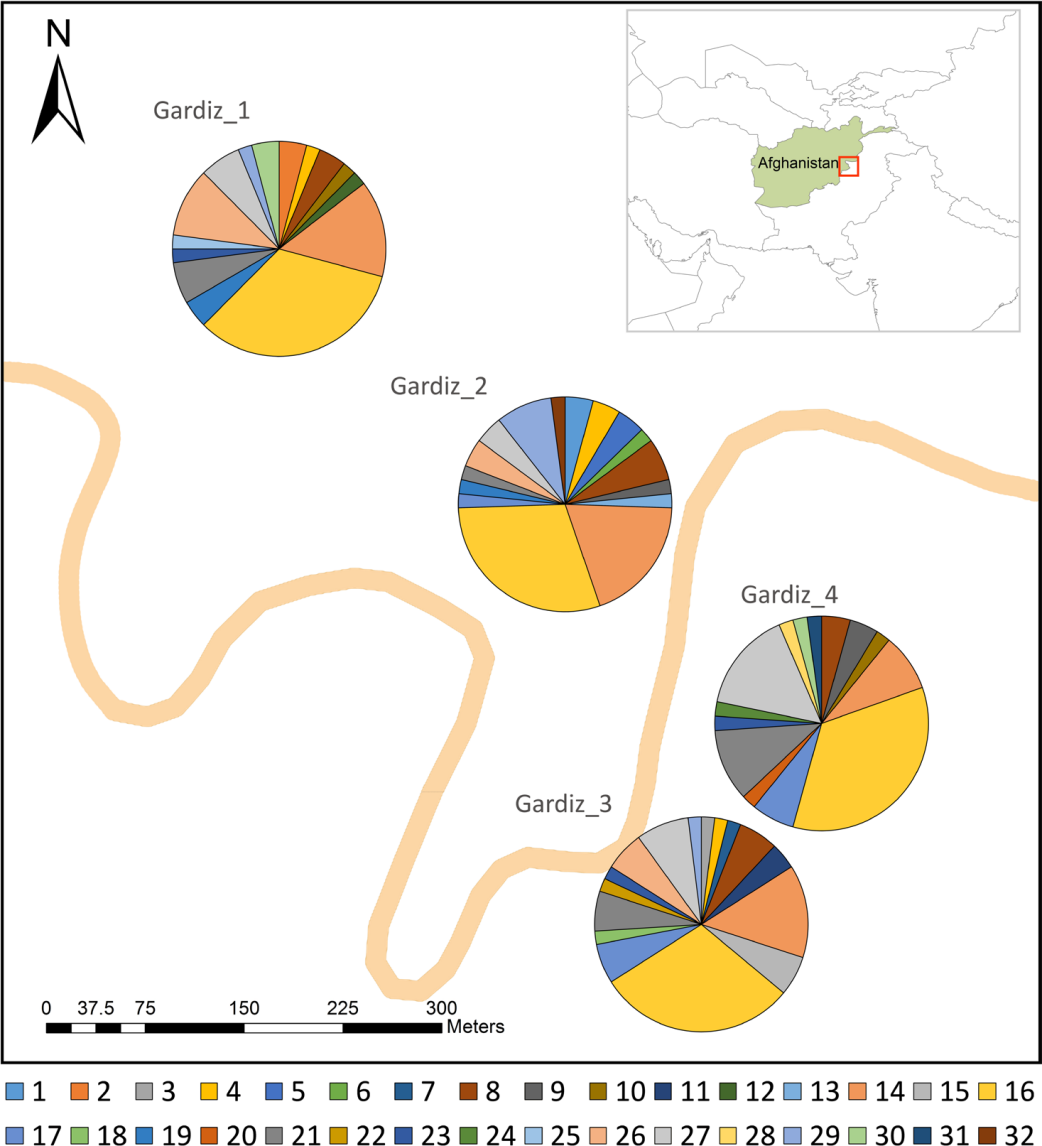
The geographical distribution of the 32 chloroplast DNA (cpDNA) haplotypes of *Pinus gerardiana* in Gardiz, Afghanistan. Each subpopulation is illustrated with a pie chart depicting the subpopulation's composition and relative frequency of haplotypes. The legend provides the color coding for each haplotype in the pie charts.

The chloroplast haplotype analysis of *P. gerardiana* in the Gardiz region revealed a total of 32 haplotypes distributed across the four subpopulations (Figure 2 and Table S2). Haplotype 16 was the most prevalent with a frequency of 31.94%. Haplotype 14 also exhibited a notable frequency, comprising 14.14% of the total haplotypes identified. On the other hand, several haplotypes appeared at lower frequencies, with some haplotypes observed only once within specific subpopulations (Table S2 and Figures S2 and S3).

### 3.2 Cross‐Species Amplification Potential

The BLAST analysis revealed significant sequence conservation across several related *Pinus* species, suggesting potential for cross‐species amplification (Table S3).

Several markers exhibited perfect matches (100% identity) with related species, indicating high evolutionary conservation of primer binding sites. Notably, markers showed extremely low E‐values (e.g., 0.0 or 9e‐152), confirming that these alignments are statistically significant and not due to random chance.

High identity and low E‐values suggest that these markers are likely to amplify successfully across multiple species. Although most markers displayed high conservation, some showed moderate identity levels (e.g., 95.45% for PGCP_5 with *P. morrisonicola*), indicating potential species‐specific loci.

### 3.3 Genetic Diversity Analysis

The genetic diversity characteristics of the developed chloroplast SSR markers are summarized in Table 3. The number of alleles (*N*_*a*_) ranged from 3.00 to 5.50 across the six markers, with the number of effective alleles (*N*_*e*_) ranging from 1.19 to 1.80. The Shannon Index (*I*) ranged from 0.34 to 0.82, indicating moderate genetic diversity. This level of diversity suggests a relatively stable population with sufficient genetic variation to potentially adapt to environmental changes. However, it is not exceptionally high, which could reflect the population's relatively small size. The total gene diversity (*H*_*t*_) ranged from 0.164 (PGCP_22) to 0.476 (PGCP_18), highlighting varying levels of polymorphism among the markers. Compared to other conifer species, these values are in a moderate range with expectations for species with limited seed dispersal and habitat specialization (Echt et al. 1998).

**TABLE 3:** Genetic characteristics of chloroplast SSR markers developed in *Pinus gerardiana* .

	cpSSR	*N*_*a*_	*N*_*e*_	*I*	*H*_*t*_
1	PGCP_2	4.00	1.19	0.36	0.168
2	PGCP_5	3.00	1.53	0.64	0.375
3	PGCP_16	5.50	1.29	0.52	0.249
4	PGCP_18	4.00	1.80	0.82	0.476
5	PGCP_21	4.50	1.53	0.69	0.362
6	PGCP_22	3.00	1.19	0.34	0.164

Abbreviations: *H*_*t*_, total gene diversity; *I*, Shannon Index; *N*_*a*_, number of alleles; *N*_*e*_, number of effective alleles.

Young and old cohorts showed high levels of genetic diversity (Table 4). The number of haplotypes (*A*) was 27 for the young cohort and 24 for the old cohort. In terms of private haplotypes (*P*), the young and old cohorts had 8 and 5 private haplotypes, respectively, with a very low frequency of each haplotype (unique occurrence). The effective number of haplotypes (*N*_*e*_) was 6.77 and 7.10 for the young and old cohorts, respectively. The haplotype richness (*R*_*h*_) was 24.12 in the young cohort and 23.00 in the old cohort. Gene diversity (*H*_*d*_) was recorded at 0.861 for the young cohort and 0.869 for the old cohort.

**TABLE 4:** Genetic variation based on haplotypes for young and old trees in *Pinus gerardiana* population from Gardiz, Afghanistan.

Cohorts	*N*	*A*	*P*	*N*_*e*_	*R*_*h*_	*H*_*d*_
Young	101	27.00	8.00	6.77	24.12	0.861
Old	90	24.00	5.00	7.10	23.00	0.869
Total	191	32.00	—	6.97	31.00	0.861

Abbreviations: *A*, number of haplotypes detected in each population; *H*_*d*_, haplotype diversity; *N*, sample size; *N*_*e*_, effective number of haplotypes; *P*, number of private haplotypes; *R*_*h*_, haplotype richness.

The genetic differentiation between subpopulations was assessed using both *F*_*st*_ and Jost's D. The *F*_*st*_ value (0.001) was very low (ranged from 0.002 to 0.004, Table S4), suggesting negligible genetic differentiation between age cohorts and subpopulations. The overall Jost's D value across all pairwise subpopulation comparisons was 0.005, indicating very low genetic differentiation as well.

### 3.4 Fine‐Scale Spatial Genetic Structure (FSGS)

**FIGURE 3:**
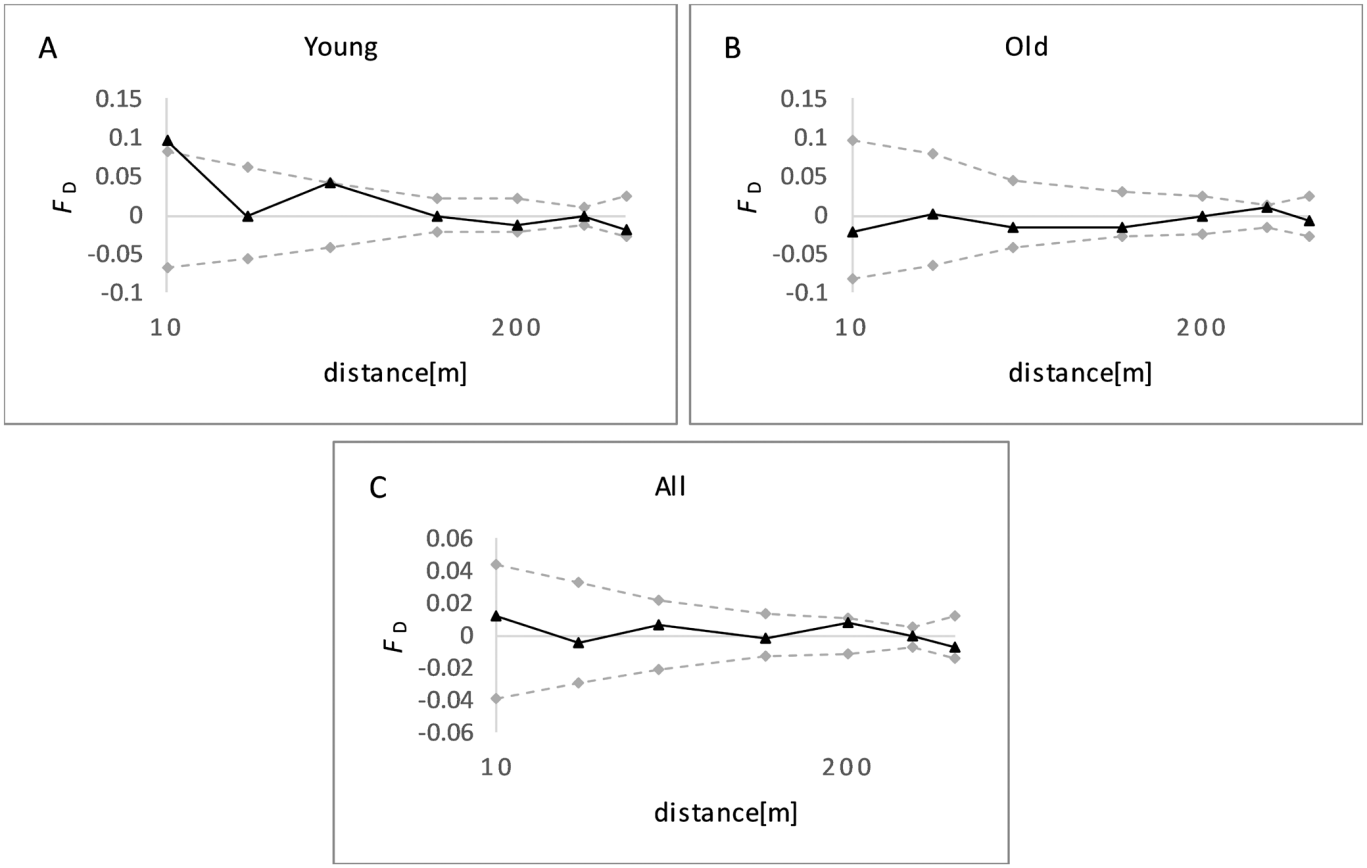
Spatial genetic structure in young (A), old (B) cohorts, and all individuals (C) of *Pinus gerardiana* using chloroplast SSRs. The kinship coefficient per distance class, *F*_D_, was plotted against the logarithm of geographical distances between individuals.

The FSGS indicated a significant family structure in the young cohort (Table 5 and Figure 3). The multilocus kinship coefficient (*F*_*1*_) between individuals of the first distance class was 0.095 for young trees and −0.021 for old trees, indicating an unrelated relationship. Regression analysis revealed a significant negative relationship between genetic relatedness and spatial distance for young trees (*b*_*f*_ = −0.010, *p* value = 0.041); however, no significant spatial genetic structure was observed for old trees (*b*_*f*_ = 0.004, *p* = 0.428). Furthermore, the *Sp* value for the young cohort (0.011) was higher than the old cohort (−0.004) and for the whole population, the *Sp* value was 0.002 (Table 5).

**TABLE 5:** Characterization of the fine‐scale spatial genetic structure using six chloroplast microsatellite markers in *Pinus gerardiana* in Gardiz, Afghanistan, for young and old trees.

	*F*_1_	*b*_*f*_	*Sp*	*p* value (*b*_*f*_)
Young	0.095	−0.010	0.011	0.041
Old	−0.021	0.004	−0.004	0.428
All	0.011	−0.002	0.002	0.228

Abbreviations: *b*_*f*_, regression slope of *F*_ij_ on natural log distance; *F*_1_, multilocus kinship coefficient between individuals of the first distance class (Loiselle et al. 1995); *Sp*, quantification of the fine‐scale SGS.

## 4 Discussion

### 4.1 Haplotype Distribution

The distribution of chloroplast haplotypes within the *P. gerardiana* population in Gardiz provides insights into the history of the study population. The high frequency of haplotype 16 possibly indicates a historical expansion after a significant population bottleneck (Provan et al. 2001; Zhang and Hewitt 2003). This bottleneck would have drastically reduced genetic diversity, leaving haplotype 16 as the predominant genetic variant in the surviving population.

The observed distribution of haplotypes across different subpopulations suggests a relatively homogenous genetic structure within the Gardiz region. This finding aligns with our previous study (Moosavi et al. 2022) based on EST‐SSR markers for this wind‐pollinated species, where gene flow by pollen contributes to the maintenance of genetic connectivity among populations. The comparatively high number of eight private haplotypes in the seedling generation suggests significant pollen influx and connectivity among populations.

In this study, we used cpSSRs to investigate paternally inherited genetic variation, as chloroplast DNA is passed down via pollen without recombination (Provan et al. 2001). Identical haplotypes among individuals suggest a shared paternal lineage rather than clonal reproduction. Our data do not provide strong evidence for clonality. Only two closely spaced paired individuals were identified as clones based on nuclear SSR and cpSSR genotypes.

### 4.2 Cross‐Amplification and Evolutionary Conservation

The BLAST analysis showed that the cpSSR markers for *P. gerardiana* are highly conserved across related species like *P. bungeana* , *P. squamata* , and *P. koraiensis* , with high identity (up to 100%) and low E‐values, indicating strong evolutionary conservation. These markers are likely to amplify successfully across species, making them useful for comparative genetic studies. Some markers showed slightly lower identity, suggesting species‐specific variations that could aid in species differentiation. These findings demonstrate the versatility of the markers for genetic research in *Pinus* species.

### 4.3 Genetic Diversity

While both age cohorts exhibited comparatively high levels of haplotype diversity, the old cohort showed slightly higher values; however, these differences were not statistically significant, indicating similar levels of genetic diversity across age cohorts. The genetic (haplotype) diversity observed in the Gardiz population of *P. gerardiana* (0.861) aligns with the diversity levels found in other conifer species. For example, Maritime pine ( *Pinus pinaster* Ait.) exhibits a mean haplotype diversity of 0.870 across 12 Portuguese populations (Ribeiro et al. 2001). Similarly, Scots pine ( *Pinus sylvestris* L.) shows a haplotype diversity of 0.750 in 23 populations in Lithuania (Kavaliauskas et al. 2022). In addition, Brutia pine ( *Pinus brutia* Ten.) has a haplotype diversity of 0.840 across 35 populations, while Aleppo pine ( *Pinus halepensis* Mill.) demonstrates a haplotype diversity of 0.710 across 21 populations, covering a wide range of their distribution (Yusuf et al. 2023).

Considering haplotype richness, both cohorts have similar levels, with the young cohort having a slightly higher value than the old cohort. The slightly higher haplotype richness in young cohorts may reflect recent genetic contributions from diverse parental individuals, ensuring the gene flow in the population.

The very low *F*_*st*_ (0.001) and Jost's D (0.005) values indicate that there is almost no genetic differentiation between the age cohorts (Nei 1973; Wright 1949) reflecting the transfer of genetic information from old to young cohort (Slatkin 1987). While this study focused on a single population, further research involving multiple populations across the species' range in Afghanistan would be necessary to assess the extent of genetic connectivity and potential isolation between populations. High gene flow can contribute to the resilience and adaptability of populations by spreading beneficial genetic variations and reducing the risk of inbreeding depression (Tallmon et al. 2004).

### 4.4 Fine‐Scale Spatial Genetic Structure (FSGS)

The FSGS analysis revealed a weak but significant family structure and relatedness in the young cohort of *P. gerardiana* , whereas no significant spatial genetic structure was observed in the old cohort. These findings suggest distance‐dependent gene flow by pollen.

These results are in accordance with our previous work using EST‐SSRs to characterize the *P. gerardiana* samples. Notably, using EST‐SSRs, a significant family structure was detected in young trees (Moosavi et al. 2022), indicative of localized pollen and seed dispersal patterns within this demographic subset. This disparity in FSGS patterns between age cohorts aligns with previous research findings in other plant species, where varying degrees of spatial genetic structure have been observed across different life stages (Berens et al. 2014; Kitamura et al. 2018).

Additionally, forest fragmentation can impede pollen flow between tree populations (Yang et al. 2015), as landscape elements between the fragments may act as barriers to pollen movement. Furthermore, the density and distribution of trees influence pollen dispersal patterns (Ghazoul 2005), with denser populations facilitating more effective pollen flow. These factors could reduce the distance over which pollen can travel, potentially leading to restricted gene flow and influencing the genetic structure of the population. In low‐density populations, as observed in *P. gerardiana* in Gardiz, longer pollen dispersal distances may occur, but the limited number of effective mates and potential isolation of individuals could still result in reduced genetic diversity and increased inbreeding (Moosavi et al. 2022). Consequently, the combination of fragmentation, low population density, and poor pollen flow could have significant long‐term effects on the genetic health and viability of the species (Sánchez‐Robles et al. 2014).

### 4.5 Implications for Conservation and Management

The cpSSR markers developed in this study offer a powerful tool for future genetic studies and conservation planning. By identifying haplotype diversity and gene flow by pollen, these markers can help prioritize areas for conservation, guide reforestation efforts, and monitor genetic diversity over time. This information is critical for ensuring the long‐term survival of *P. gerardiana* and for implementing effective management strategies in response to habitat fragmentation, climate change, and anthropogenic disturbances.

The findings from this study highlight the importance of maintaining genetic diversity and gene flow in *P. gerardiana* populations. The moderate genetic diversity in both age cohorts and high gene flow observed suggest a stable population, but the limited seed dispersal capacity and sparse natural regeneration imply that conservation efforts must focus on maintaining connectivity across populations from other regions. Forest fragmentation, which can restrict pollen and seed dispersal, may further exacerbate the risks of reduced genetic diversity and result in increased inbreeding. Therefore, protecting and managing habitat corridors to ensure effective gene flow between populations is crucial.

The significant role of *Nucifraga multipunctata* (large‐spotted nutcracker) as a seed disperser should be emphasized in conservation strategies. Efforts to protect this bird species, along with the forest habitats it relies on, will promote successful regeneration of *P. gerardiana* . It is also recommended to expand research beyond the Gardiz region, including multiple populations across Afghanistan and neighboring regions, such as those in Pakistan near the Afghanistan border, to gain a comprehensive understanding of genetic connectivity. Such research could guide future conservation strategies to ensure the long‐term resilience and survival of *P. gerardiana* in the face of environmental change and anthropogenic pressures.

## 5 Conclusion

Our study provides the first comprehensive assessment of genetic diversity in *P. gerardiana* populations from Afghanistan using novel chloroplast microsatellite markers (cpSSRs). The high levels of genetic diversity, weak spatial genetic structure, and high number of private haplotypes in the young generation suggest efficient gene flow by pollen. These findings can provide a foundation for future genetic studies and the development of conservation strategies aimed at preserving the genetic resources of this ecologically and economically valuable species.

## Author Contributions

**Sayed Jalal Moosavi:** conceptualization (equal), data curation (equal), formal analysis (lead), investigation (equal), methodology (equal), resources (equal), software (equal), validation (equal), visualization (lead), writing – original draft (lead), writing – review and editing (equal). **Markus Mueller:** conceptualization (equal), data curation (equal), investigation (equal), methodology (equal), validation (equal), writing – review and editing (equal). **Oliver Gailing:** conceptualization (equal), data curation (equal), investigation (equal), methodology (equal), project administration (lead), resources (equal), supervision (lead), validation (equal), writing – review and editing (equal).

## Conflicts of Interest

The authors declare no conflicts of interest.

## Supporting information


Data S1.

## Data Availability

cpSSR data, GPS coordinates, and age class of each individual were deposited in the Göttingen Research Online repository (https://doi.org/10.25625/OCBGBS).
