# Supplementary material for: Development of New Chloroplast Microsatellites for Pinus gerardiana and their Application in Genetic Diversity Analyses
Source: Ecol Evol. 2025 Apr 1;15(4):e71185. doi: 10.1002/ece3.71185 (PMC11961383; doi:10.1002/ece3.71185)
Supplement: Supplementary file 1 — Data S1. [file ECE3-15-e71185-s001.docx]

Development of new chloroplast microsatellites for *Pinus gerardiana* and their application in genetic diversity analyses

Sayed Jalal Moosavi^1^, Markus Mueller^1^, Oliver Gailing^1^

^1^Forest Genetics and Forest Tree Breeding, University of Göttingen, Büsgenweg 2, 37077 Göttingen, Germany

Contact information: Oliver Gailing ([ogailin@gwdg.de](mailto:ogailin@gwdg.de), +49-551-3923536), Markus Müller ([mmuellef@gwdg.de](mailto:mmuellef@gwdg.de), +49-551-3928238) and Sayed Jalal Moosavi ([smoosav@gwdg.de](mailto:smoosav@gwdg.de), +49-551-399522).

Corresponding authors: Oliver Gailing and Sayed Jalal Moosavi

**Supplementary file**


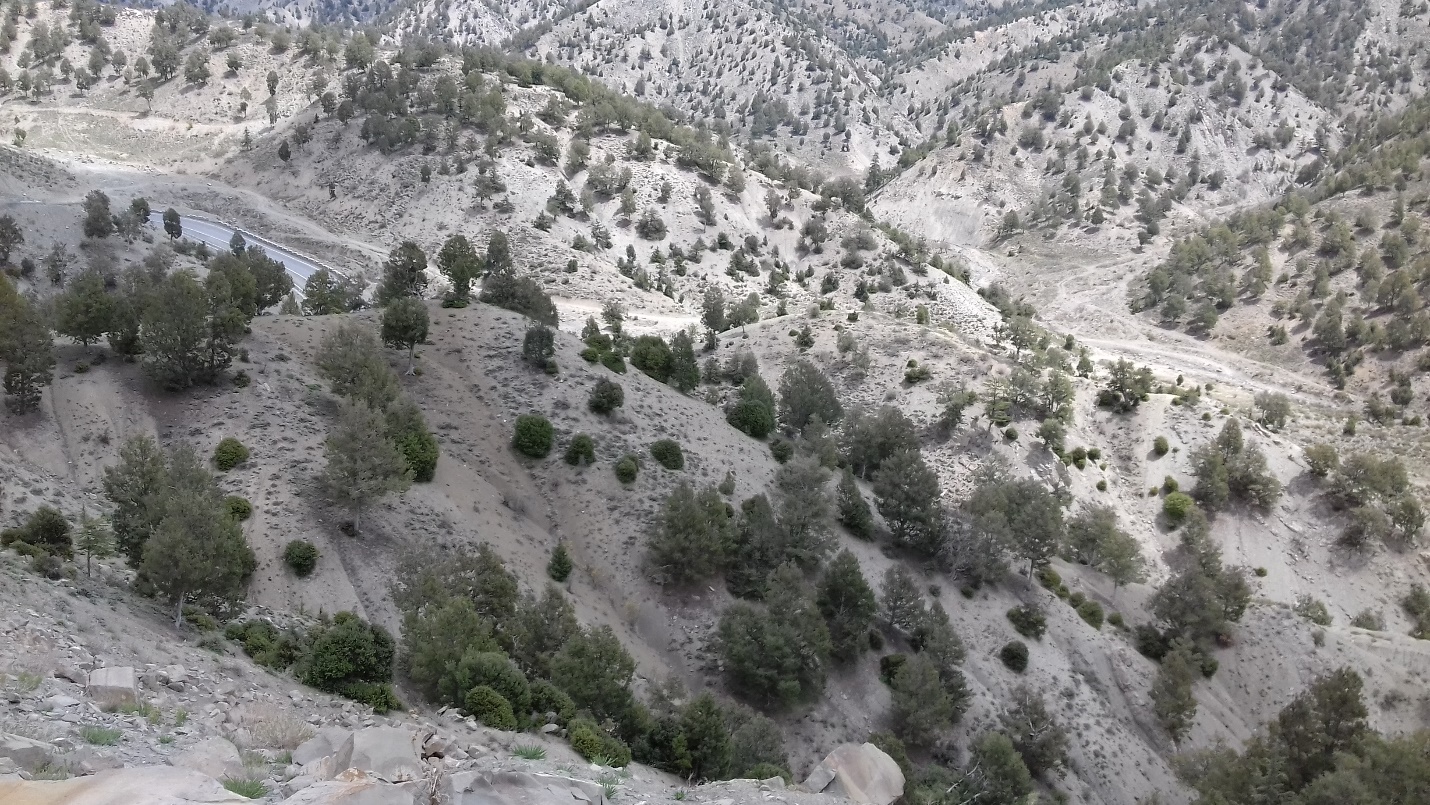


Figure S1: View of the *Pinus gerardiana* pine forest near Gardiz city in Paktia province, Afghanistan, showing the sparse tree density and the rugged mountains of the region. This area served as the sampling location for the study.

Table S1: Primers developed for *Pinus gerardiana* chloropalst SSR Markers.

| **Marker name** | **Motif** | **Start Position** | **End Position** | **Sequence (5'->3')*** | **Tm (°C)** | **Product size (bp)** |
| --- | --- | --- | --- | --- | --- | --- |
| PGCP_1 | (T)_12_ | 13132 | 13143 | F: TGCTTGCCCTATCCATTCCC | 60 | 322 |
|  |  |  |  | R: TTGCGAGACAACCAGAAGCA | 60 |  |
| PGCP_2 | (A)_24_ | 29333 | 29356 | F: TTATCCATCGGCCCAGTTCC | 60 | 190 |
|  |  |  |  | R: CGATCTTTTGTCCAACCAACCC | 60 |  |
| PGCP_3 | (A)_10_ | 36199 | 36208 | F: TGCCTTTCAGTCAAGTGATGAGT | 60 | 102 |
|  |  |  |  | R: TCTGTGGTTCCTTCGACAGAG | 59 |  |
| PGCP_4 | (T)_13_ | 36461 | 36473 | F: TGGCTTACAAAATAAAAGAGGAGG | 57 | 130 |
|  |  |  |  | R: GAGCAGACAAGTAAGGGGCA | 60 |  |
| PGCP_5 | (A)_12_tatactcggg(A)_12_ | 39019 | 39052 | F: TCACTTGTCAGTTCTGTCCGA | 59 | 173 |
|  |  |  |  | R: CCTTATGCTCCGTCTCACCC | 60 |  |
| PGCP_6 | (TTTA)_5_ | 41073 | 41092 | F: TTCGTTGTTCCTTTCCCCGT | 60 | 273 |
|  |  |  |  | R: TGCCGAACCTAAAGCGTACA | 60 |  |
| PGCP_7 | (T)_23_ | 44679 | 44701 | F: ACCAAGGTGGTCACTTCCAT | 59 | 187 |
|  |  |  |  | R: AAGTGCGGAAACCCCAAGAA | 60 |  |
| PGCP_8 | (T)_10_ | 47860 | 47869 | F: GGACCAGAGCAGGCAGATTA | 59 | 165 |
|  |  |  |  | R: TTGACTGCACGATGGATTGC | 59 |  |
| PGCP_9 | (T)_12_ | 51659 | 51670 | F: CAGGAATAGCTCCGCTCTTGA | 60 | 316 |
|  |  |  |  | R: TGTTATCGAGCTAGACCCGGA | 60 |  |
| PGCP_10 | (A)_14_ | 54180 | 54193 | F: CCTGGATGGGGAACTACTGC | 60 | 301 |
|  |  |  |  | R: TCAACAAGTCGCACACCCAT | 60 |  |
| PGCP_11 | (T)_12_ | 57167 | 57178 | F: GGTTAGGTATTGGGGCAGCAT | 60 | 311 |
|  |  |  |  | R: GGGTTCATAAAATGGTGGGTTTCA | 60 |  |
| PGCP_12 | (A)_10_ | 60447 | 60456 | F: TTCGGGCTCCACTGTTATCC | 59 | 247 |
|  |  |  |  | R: GAGTATCCGAAACCGTCGCT | 60 |  |
| PGCP_13 | (T)_14_ | 63268 | 63281 | F: ACCGATCATTGCGGGTACAA | 60 | 323 |
|  |  |  |  | R: TGGAGGTGGGGAAGGAAGAA | 60 |  |
| PGCP_14 | (T)_10_ | 64065 | 64074 | F: TGTCCCGATTCTCTACATCCT | 58 | 249 |
|  |  |  |  | R: GATCTGATGGTCAGAGGCGG | 60 |  |
| PGCP_15 | (T)10 | 68848 | 68857 | F: TACGATTGTTCGTCGGGTCC | 60 | 366 |
|  |  |  |  | R: AGCCGGATGCGATATTGAGA | 59 |  |
| PGCP_16 | (T)_14_ | 71336 | 71349 | F: CCGGAGAATACAGGGCGTTA | 59 | 327 |
|  |  |  |  | R: TTCCGCATATTCCCCTTCCG | 60 |  |
| PGCP_17 | (AT)_6_ | 72873 | 72884 | F: TGGATGGCTTTGATATGGGACA | 59 | 229 |
|  |  |  |  | R: TCGATCTGAGGCAGGTGTTC | 59 |  |
| PGCP_18 | (T)_16_ | 79112 | 79127 | F: TGAGTGTGAGAGGAGAGGGAA | 60 | 202 |
|  |  |  |  | R: GGTTTTCAAGACCGGAGCCA | 61 |  |
| PGCP_19 | (A)_12_ | 85295 | 85306 | F: CCCAGGTCGGAACAAGTTGA | 60 | 168 |
|  |  |  |  | R: TAATAGCCCATCGTGCAGGC | 60 |  |
| PGCP_20 | (A)_10_ | 100615 | 100624 | F: GTCGATCCGCGATGTTGAGA | 60 | 153 |
|  |  |  |  | R: AACGGAACGAACAGATTGGT | 58 |  |
| PGCP_21 | (T)_14_ | 100834 | 100847 | F: CGAAATGGTCGGAACGAATCA | 59 | 277 |
|  |  |  |  | R: GCCACAAACCCCTTTGGGAT | 61 |  |
| PGCP_22 | (T)_11_ | 101716 | 101726 | F: GCGCAGTATGGGTCTAGCTT | 60 | 183 |
|  |  |  |  | R: AACCCGCAGATACAGGCAAA | 60 |  |
| PGCP_23 | (T)_11_ | 104789 | 104799 | F: ACTTTTCGAATGACCGGTGGA | 60 | 153 |
|  |  |  |  | R: ACCCCATTGTTTCTTATGGCAG | 59 |  |
| PGCP_24 | (A)_15_ | 107059 | 107073 | F: GAGAAGGAAGAGGCAATCCCA | 59 | 234 |
|  |  |  |  | R: TGCACCTCATACGGCTTCTC | 60 |  |
| PGCP_25 | (AT)_10_ | 109830 | 109849 | F: TTGAAAAAGAAAGGATGTTCAGTCA | 58 | 258 |
|  |  |  |  | R: TTGAGTCTCGCGTGTCTACC | 59 |  |
| PGCP_26 | (T)_10_ | 110493 | 110502 | F: TGGTCGAAATCTCGGAGTGAG | 60 | 176 |
|  |  |  |  | R: GCAGTAGGGTCGTGGGAATC | 60 |  |
| PGCP_27 | (T)_10_ | 116837 | 116846 | F: AGCTTTATAAGGCGCTCCCG | 60 | 236 |
|  |  |  |  | R: TGGTTGACACGAGCTTTCCA | 60 |  |

*F primer, Forward primer; R primer, Reverse primer

Table S2: *P. gerardiana* chloroplast haplotypes and their frequencies observed in different subpopulations (Gardiz_1, Gardiz_2, Gardiz_3 and Gardiz_4) in Gardiz, Afghanistan.

| **Haplotype ID** | **Marker name** | | | | | | **Subpopulation** | | | |  |  |
| --- | --- | --- | --- | --- | --- | --- | --- | --- | --- | --- | --- | --- |
|  | **PGCP_2** | **PGCP_5** | **PGCP_16** | **PGCP_18** | **PGCP_21** | **PGCP_22** | **Gardiz_1** | **Gardiz_2** | **Gardiz_3** | **Gardiz_4** | **N** | **Frequency %** |
| 16 | 208 | 198 | 359 | 231 | 312 | 209 | 16 | 14 | 15 | 16 | 61 | 31.94 |
| 14 | 208 | 198 | 359 | 230 | 312 | 209 | 7 | 9 | 7 | 4 | 27 | 14.14 |
| 27 | 208 | 199 | 359 | 231 | 312 | 209 | 3 | 2 | 4 | 7 | 16 | 8.38 |
| 21 | 208 | 198 | 359 | 232 | 313 | 209 | 3 | 1 | 3 | 5 | 12 | 6.28 |
| 8 | 208 | 197 | 359 | 231 | 312 | 209 | 2 | 3 | 3 | 2 | 10 | 5.24 |
| 26 | 208 | 198 | 361 | 231 | 312 | 209 | 5 | 2 | 3 | - | 10 | 5.24 |
| 17 | 208 | 198 | 359 | 231 | 313 | 209 |  | 1 | 3 | 3 | 7 | 3.66 |
| 29 | 209 | 198 | 359 | 231 | 312 | 208 | 1 | 4 | 1 | - | 6 | 3.14 |
| 4 | 208 | 197 | 359 | 229 | 311 | 210 | 1 | 2 | 1 | - | 4 | 2.09 |
| 9 | 208 | 197 | 359 | 231 | 313 | 209 | - | 1 | - | 2 | 3 | 1.57 |
| 15 | 208 | 198 | 359 | 230 | 312 | 210 | - | - | 3 | - | 3 | 1.57 |
| 19 | 208 | 198 | 359 | 231 | 314 | 209 | 2 | 1 | - | - | 3 | 1.57 |
| 23 | 208 | 198 | 360 | 231 | 312 | 209 | 1 | - | 1 | 1 | 3 | 1.57 |
| 30 | 209 | 198 | 359 | 231 | 312 | 209 | 2 | - | - | 1 | 3 | 1.57 |
| 1 | 205 | 198 | 360 | 231 | 312 | 209 | - | 2 | - | - | 2 | 1.05 |
| 2 | 207 | 198 | 359 | 230 | 312 | 209 | 2 |  | - | - | 2 | 1.05 |
| 5 | 208 | 197 | 359 | 230 | 312 | 209 | - | 2 | - | - | 2 | 1.05 |
| 10 | 208 | 198 | 355 | 231 | 312 | 209 | 1 | - | - | 1 | 2 | 1.05 |
| 11 | 208 | 198 | 358 | 231 | 312 | 209 | - | - | 2 | - | 2 | 1.05 |
| 3 | 207 | 198 | 359 | 231 | 312 | 209 | - | - | 1 | - | 1 | 0.52 |
| 6 | 208 | 197 | 359 | 231 | 310 | 209 | - | 1 | - | - | 1 | 0.52 |
| 7 | 208 | 197 | 359 | 231 | 311 | 209 | - | - | 1 | - | 1 | 0.52 |
| 12 | 208 | 198 | 359 | 230 | 310 | 210 | 1 | - | - | - | 1 | 0.52 |
| 13 | 208 | 198 | 359 | 230 | 311 | 210 | - | 1 | - | - | 1 | 0.52 |
| 18 | 208 | 198 | 359 | 231 | 313 | 210 | - | - | 1 | - | 1 | 0.52 |
| 20 | 208 | 198 | 359 | 232 | 312 | 209 | - | 0 | - | 1 | 1 | 0.52 |
| 22 | 208 | 198 | 359 | 232 | 314 | 209 | - | - | 1 | - | 1 | 0.52 |
| 24 | 208 | 198 | 360 | 231 | 313 | 209 | - | - | - | 1 | 1 | 0.52 |
| 25 | 208 | 198 | 360 | 231 | 314 | 209 | 1 | - | - | - | 1 | 0.52 |
| 28 | 208 | 199 | 359 | 231 | 313 | 209 | - | - | - | 1 | 1 | 0.52 |
| 31 | 209 | 198 | 359 | 231 | 313 | 209 | - | - | - | 1 | 1 | 0.52 |
| 32 | 209 | 199 | 359 | 231 | 312 | 209 | - | 1 | - | - | 1 | 0.52 |


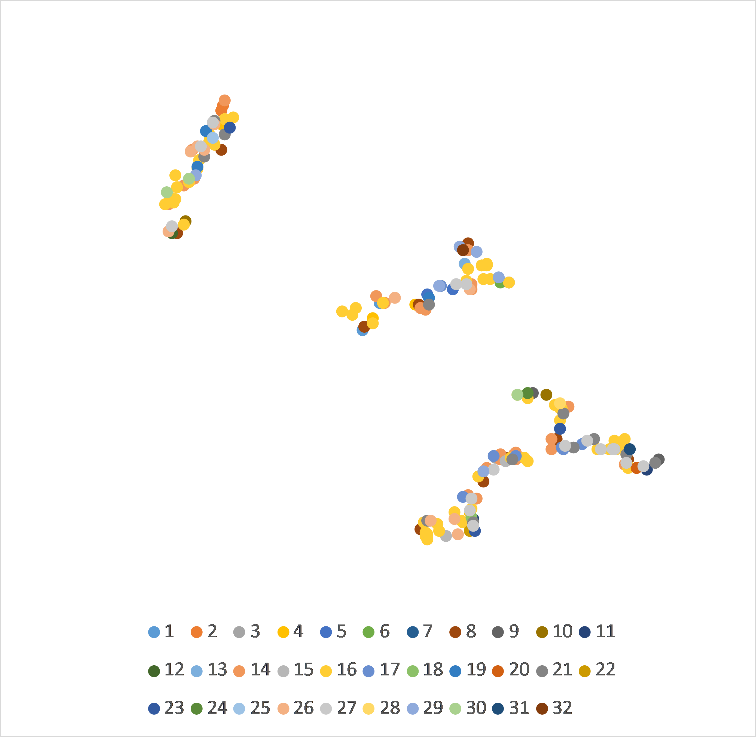


Figure S2: Haplotype distribution among *Pinus gerardiana* individual trees based on cpSSR markers. Each dot represents a tree, with different colors indicating the presence of distinct haplotypes.


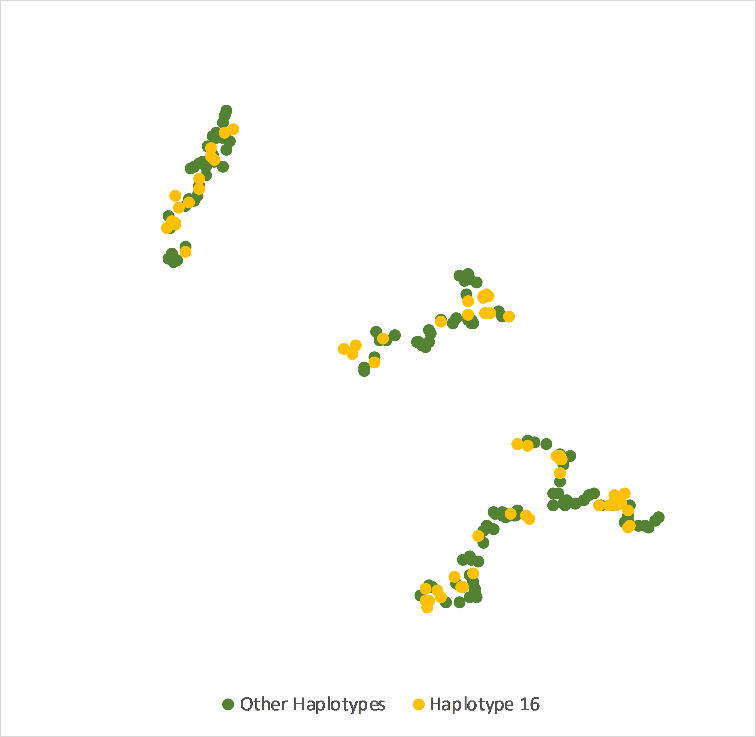


Figure S3: Geographic distribution of Haplotype 16 compared to other haplotypes. Trees with Haplotype 16 are marked in yellow, while those with other haplotypes are marked in green.

Table S3: BLAST results for cpSSR markers, highlighting the best match species, percent identity, and E-value.

| **Marker** | **Best match species** | **Query cover (%)** | **% Identity** | **E-value** | **Remarks** |
| --- | --- | --- | --- | --- | --- |
| PGCP_1 | *P. bungeana* | 100 | 98.68 | 9e-152 | Moderate conservation, cross-species potential. |
| PGCP_2 | *P. squamata* | 100 | 99.47 | 1e-94 | Highly conserved, cross-species potential. |
| PGCP_3 | *P. koraiensis* | 100 | 97.56 | 2e-33 | Moderate conservation. |
| PGCP_4 | *P. monticola* | 100 | 98.20 | 2e-49 | Moderate conservation. |
| PGCP_5 | *P. morrisonicola* | 100 | 95.45 | 4e-63 | Moderate conservation, species-specific potential. |
| PGCP_6 | *P. dabeshanensis* | 91 | 99.00 | 5e-99 | Highly conserved, cross-species potential. |
| PGCP_7 | *P. Pumila* | 100 | 100.00 | 3e-84 | Highly conserved, perfect match. |
| PGCP_8 | *P. bungeana* | 100 | 99.32 | 2e-70 | Highly conserved. |
| PGCP_9 | *P. bungeana* | 100 | 99.66 | 2e-153 | Highly conserved. |
| PGCP_10 | *P. squamata* | 100 | 100.00 | 2e-147 | Highly conserved, perfect match. |
| PGCP_11 | *P. squamata* | 100 | 100.00 | 1e-150 | Highly conserved, perfect match. |
| PGCP_12 | *P. morrisonicola* | 100 | 100.00 | 2e-117 | Highly conserved, perfect match. |
| PGCP_13 | *P. morrisonicola* | 100 | 100.00 | 1e-159 | Highly conserved, perfect match. |
| PGCP_14 | *P. ayacahuite* | 100 | 99.57 | 2e-116 | Highly conserved. |
| PGCP_15 | *P. koraiensis* | 100 | 100.00 | 0.0 | Highly conserved, perfect match. |
| PGCP_16 | *P. monticola* | 78 | 99.58 | 7e-123 | Highly conserved. |
| PGCP_17 | *P. bungeana* | 100 | 100.00 | 2e-107 | Highly conserved, perfect match. |
| PGCP_18 | *P. squamata* | 100 | 100.00 | 2e-92 | Highly conserved, perfect match. |
| PGCP_19 | *P. lambertiana* | 100 | 99.33 | 2e-71 | Highly conserved. |
| PGCP_20 | *P. strobus* | 100 | 99.25 | 9e-64 | Highly conserved. |
| PGCP_21 | *P. wangii* | 100 | 99.61 | 2e-132 | Highly conserved. |
| PGCP_22 | *P. bungeana* | 100 | 100.00 | 5e-82 | Highly conserved, perfect match. |
| PGCP_23 | *P. monophylla* | 100 | 100.00 | 5e-61 | Highly conserved, perfect match. |
| PGCP_24 | *P. koraiensis* | 100 | 99.53 | 1e-108 | Highly conserved. |
| PGCP_25 | *P. bungeana* | 100 | 99.16 | 1e-119 | Highly conserved. |
| PGCP_26 | *P. cembra* | 100 | 100.00 | 4e-78 | Highly conserved, perfect match. |
| PGCP_27 | *P. wangii* | 100 | 99.54 | 1e-109 | Highly conserved. |

Table S4: Pairwise *F*_st_ values between four subpopulations of *Pinus gerardiana* in Gardiz, Afghanistan.

|  | Gardiz_1 | Gardiz_2 | Gardiz_3 | Gardiz_4 |
| --- | --- | --- | --- | --- |
| Gardiz_1 | 0.000 |  |  |  |
| Gardiz_2 | 0.002 | 0.000 |  |  |
| Gardiz_3 | 0.002 | 0.002 | 0.000 |  |
| Gardiz_4 | 0.003 | 0.004 | 0.002 | 0.000 |
